# What We Owe to Experiments on Animals

**Published:** 1904-02-13

**Authors:** Stephen Paget


### HOSPITAL CLINICS AND MEDICAL PROGRESS.

### WHAT WE OWE TO EXPERIMENTS ON ANIMALS.

By STEPHEN PAGET.

(*Concluded from page* 334.)

### *III.—THE ACT RELATING TO EXPERIMENTS ON ANIMALS.* (*Act* 39 *and* 40, *Vict. c.* 77).

IN 1875, a Royal Commission inquired into the whole subject of experiments on animals. In 1876, the present Act was passed. The evidence before the Commission was all, or nearly all, about physiology. Hardly a word was said about bacteriology; there was no bacteriology to say anything about. The lawyers who drafted the Act may have been very good lawyers, but they were very bad prophets. They did not foresee the time when six out of seven experiments would be bacteriological, which is the case now. Let us take a look at the Act. The text of the Act, and the annual reports by the inspectors under the Act, are to be had for a few pence from Eyre and Spottiswoode, East Harding Street, Fleet Street, E.C.

Let us suppose that a man wishes to inoculate some animals in the hope of helping to find the bacillus of measles. He applies to the Home Office, and receives two blank forms: one for a License, the other for Certificate A. These he must fill up with a description of the proposed experiments, and must get them signed by two authorities. The number of those who are qualified under the Act to sign these forms is very small; some are presidents of learned societies, and the rest are professors of the medical sciences. Thus signed and countersigned, the forms go back to the Home Office, which submits them to the judgment of certain non-official experts whom it has accepted as its private advisers: it also imposes its own conditions and restrictions as to the number of inoculations to be made,[1] or any other rule that it may choose to enforce. If the animals to be inoculated are any of those specially protected by the Act, a special Certificate (E or F) is necessary, in addition to Certificate A. Every inoculation must be recorded and reported; every laboratory must submit to "surprise visits"; and censure or disqualification have been administered in those few, very few, cases where a man has failed to render complete obedience to the letter and the spirit of the Act. What more could be desired, even by the most vehement anti-vivisection society? One of them says that it wants more inspectors. It would not be altogether unreasonable to appoint Government Inspectors of these pestilent societies.

But let us look at the Act. Why, for the inoculation of a rabbit, is Certificate A necessary? That question takes us back to 1876, before the days of bacteriology. *There was no provision made for inoculations in the Act.* Nobody, a quarter of a century ago, was thinking of them. Then came bacteriology. What was to be done? How was the Act, drafted before inoculations, to be made to cover inoculations? They could not be called painless and put outside the scope of the Act; the prick of the needle was painless, but its consequences might not be painless. They could not be allowed under a license alone, for they were made without anæsthetics. They could not be made under a license *plus* Certificate B (the certificate which allows the animal to be kept alive after an operation done under an anæsthetic), because the experiment, in an inoculation, is not the prick of the needle, but the consequences of the inoculation; and these consequences go on till the animal recovers, or dies, or is killed—unless indeed the result is negative, and nothing happens. The only possible certificate for inoculations was Certificate A, which permits experiments to be made without anæsthetics. There was no other way out of this absurd difficulty. All inoculations had to be brought under the Act; they all had to be scheduled, somehow or other. And the result is, that the inoculation of a mouse, even though it may not so much as make the mouse feverish, must yet be reported to Government as an experiment made without anæsthetics; and false statements are distributed far and wide, with Certificate A for a text, and it may be that many subscriptions are got in this way by the societies out of credulous people.

Now we come to Certificate B. It runs thus:—"Whereas ——— has represented to us (the two authorities) that he proposes, if duly authorised under the above-mentioned Act, to perform on living animals certain experiments described below, such animals being, during the whole of the initial operation of such experiments, under the influence of some anæsthetic of sufficient power to prevent their feeling pain, we hereby certify that, in our opinion, the killing of the animal on which any such experiment is performed before it recovers from the influence of the anæsthetic administered to it would necessarily frustrate the object of such experiment."

Some years ago, the Home Office changed the wording of this certificate. The words used to be "during the whole of such experiments." They are now, as above, "during the whole of the initial operation of such experiments." The anti-vivisectionist societies attach great importance to this change of words, and say that it permits any licensee to inflict a wound under anæsthetics and then to go on torturing the animal, through the newly-made wound, without anæsthetics. It does not: it simply brings Certificate B (or B+EE) into line with Certificate A (or A+E). And if anybody thinks that Certificate B is "given away with a pound of tea" at the Home Office, and no questions asked, he is mightily mistaken.

Now we come to curari. "The substance known as urari or curare shall not for the purposes of this Act be deemed to be an anæsthetic." That is the law: nobody doubted in 1876 that Claude Bernard's vivid account of the action of curari was the truth,

[1] It is said sometimes, by this or that Anti-vivisection Society, that a single experiment may involve the use of many animals. This is absolutely false. The number of animals used does not exceed the number of experiments given in the returns to Government.

the whole truth, and nothing but the truth: and the Act, very properly, took especial care to prevent the use of curari to keep an animal quiet under pain. But we are told now, on excellent authority, that Bernard's account of the drug was not the whole truth. "It is quite true," says Professor Rüffer, "that curare in small doses has the effect of paralysing the motor nerves without affecting the nerves of sense; but in such doses as are used in the laboratory it paralyses both sets of nerves, and this has actually been proved on man, as there have been cases of accidental curare poisoning in men who recovered, and in whom sensation has been totally abolished, while the action of the drug was apparent." Anyhow, the use of curari instead of an anæsthetic is absolutely forbidden by the Act: and, in those few experiments where it is used (not to keep the animal quiet, but for some physiological purpose) it is always used with an anæsthetic. "It is illegal," said the Home Secretary, in 1899, "to use curare as an anæsthetic. It is often used in addition to anæsthetics, for very good reasons; and, as it does not render an anæsthetised animal sensitive, it would be absurd to forbid its use." This word *often* is vague: certainly, in the vast majority of experiments, curari is never used.

Finally we come to morphia. When we consider how many deaths occur, either by accident or by design, from an overdose of morphia, we can hardly refuse to call it an anæsthetic. And, with such doses as are given in laboratories, it may often happen that the animal dies of morphia. Except in very rare cases, animals take morphia well, and are profoundly influenced by it. Among dogs, as among human beings, there are very rare cases, where morphia fails to act. (*See Brit. Med. Jour.*, January 14th, 1899.) In such a case some other anæsthetic would be given, of the usual kind: chloroform, ether, or the A.C.E. mixture of alcohol, chloroform, and ether.

Let us now take the latest Report to Government, ordered to be printed June 8th, 1903. In Ireland, only 65 experiments were made during 1902, and all except six of them were inoculations or injections. The report for England and Scotland must be put at full length: only the tables must be omitted, for want of space. Every word of this Report should be studied.

---

REPORT.

The names of all those persons who held licenses during 1902 will be found in Tables I. and II. The total number of licensees was 319, of whom 112 performed no experiments.

The considerable increase (62) in the number of licensees during 1902, as compared with the previous year, is in part due to the circumstance that licenses, instead of terminating on December 31 in each year, as was formerly the case, are now continued until the end of February in the succeeding year, in order that the renewals, when required, may be effected after the annual returns of experiments have been made and before the licenses expire. In this way the tables contain the names of 34 licensees whose licenses expired on February 28, 1902, and were not renewed for the remainder of the year. Of these, only two performed experiments in 1902.

The names of all those "registered places" to which licensees were accredited are given in the tables. All licensees were restricted to the registered place or places specified on their licenses, with the exception of (1) those who were permitted to perform inoculation experiments in places other than a "registered place," with the object of studying outbreaks of disease among animals in remote districts, and (2) two licensees who were permitted to perform certain experiments dealing with the effects of increased atmospheric pressure, and requiring the employment of apparatus which was not available at a laboratory, at a place which was not registered; this place was open to inspection, and has been frequently visited.

Tables I. and II. afford evidence—

1. That licenses and certificates have been granted and allowed only upon the recommendation of persons of high scientific standing;

2. That the licensees are persons who, by their training and education, are fitted to undertake experimental work and to profit by it;

3. That all experimental work has been conducted in suitable places.

Table III. shows the number and the nature of the experiments performed by each licensee mentioned in Table I., specifying whether these experiments were done under the license alone or under any special certificate.

Table III. is divided into two parts, A and B, for the purpose of separating experiments which are performed without anæsthetics from experiments in which anæsthetics are used.

The total number of experiments included in Table III. (A) is 2,130.

Of these there were performed,—

| | |
|---|---|
| Under License alone [1] ... ... | 1,014 |
| " Certificate C... ... ... | 171 |
| " Certificate B... ... ... | 693 |
| " Certificate B+EE ... ... | 252 |

Table III. (B) is devoted entirely to inoculations, hypodermic injections, and some few other proceedings, performed without anæsthetics. It includes 12,776 experiments, whereof there were performed,—

| | |
|---|---|
| Under Certificate A... ... ... | 12,630 |
| " Certificate A+E ... ... | 92 |
| " Certificate A+F ... ... | 54 |

The total number of experiments is 14,906, being 3,261 more than in 1901; the increase in the

---

[1] In experiments performed under license alone, the animal must, during the whole of the experiment, be under the influence of some anæsthetic of sufficient power to prevent the animal feeling pain; and the animal must, if the pain is likely to continue after the effect of the anæsthetic has ceased, or if any serious injury has been inflicted on the animal, be killed before it recovers from the influence of the anæsthetic which has been administered.

Certificate C allows experiments to be performed, under the foregoing provisions as to the use of anæsthetics, in illustration of lectures.

Certificate B exempts the person performing the experiment from the obligation to cause the animal on which the experiment is performed to be killed before it recovers from the influence of the anæsthetic; and when the animal is a dog or a cat, Certificate EE is also necessary.

Certificate A allows experiments to be performed without anæsthetics; but when the animal on which the experiment is performed is a dog or a cat, Certificate E is also necessary.

Certificate F is required in all cases of experiments on a horse, ass, or mule.

number of experiments included in Table III. (A) is 81, and in Table III. (B), 3,180.

All experiments involving a serious operation are placed in Table III. (A). The larger part of the experiments included in this table, viz., all performed under license alone, and under Certificate C, 1,185 in number, are unattended by pain, because the animal is kept under an anæsthetic during the whole of the experiment, and must, if the pain is likely to continue after the effect of the anæsthetic has ceased or if any serious injury has been inflicted on the animal, be killed before it recovers from the influence of the anæsthetic.

In the experiments performed under Certificate B, or B linked with EE, 945 in number, the initial operations are performed under anæsthetics, from the influence of which the animals are allowed to recover. The operations are performed aseptically, and the healing of the wounds, as a rule, takes place without pain. *If the antiseptic precautions fail, and suppuration occurs, the animal is required to be killed.* It is generally essential for the success of these experiments that the wounds should heal cleanly and the surrounding parts remain in a healthy condition. *Experiments involving the removal of important organs, and even of parts of the brain, are performed without causing pain to the animals; and after the section of a part of the nervous system, the resulting degenerative changes are painless.*

In the event of a subsequent operation being necessary in an experiment performed under Certificate B, or B linked with EE, a condition is attached to the license requiring all operative procedures to be carried out under anæsthetics of sufficient power to prevent the animal feeling pain.

*In no case has a cutting operation more severe than a superficial venesection been allowed to be performed without anæsthetics.*

The experiments included in Table III. (B), 12,776 in number, are all performed without anæsthetics. They are mostly inoculations, but a few are feeding experiments, or the administration of various substances by the mouth, or the abstraction of a minute quantity of blood for examination. In no instance has a certificate dispensing with the use of anæsthetics been allowed for an experiment involving a serious operation. *Inoculations into deep parts, involving a preliminary incision in order to expose the part into which the inoculation is to be made, are required to be performed under anæsthetics,* and are therefore placed in Table III. (A).

It will be seen that the operative procedures in experiments performed under Certificate A, without anæsthetics, are only such as are attended by no considerable, if appreciable, pain. The certificate is, in fact, not required to cover these proceedings, but to allow of the subsequent course of the experiment. The experiment lasts during the whole period from the administration of the drug, or injection, until the animal recovers from the effects, if any, or dies, or is killed, possibly extending over several days, or even weeks. The substance administered may give rise to poisoning, or set up a condition of disease, either of which may lead to a fatal termination. To administer to an animal such a poison as diphtheria toxin for example, or to induce such a disease as tuberculosis, although it may not be accompanied by acute suffering, is held to be a proceeding "calculated to give pain," and therefore experiments of the kind referred to come within the scope of the Act 39 & 40 Vict., c. 77. The Act provides that, unless a special certificate be obtained, the animal must be kept under an anæsthetic during the whole of the experiment; and it is to allow the animal to be kept without an anæsthetic during the time required for the development of the results of the administration that Certificate A is given and allowed in these cases.

It must not be assumed that the animal is in pain during the whole of this time. In cases of prolonged action of an injected substance, even when ending fatally, the animal is generally apparently well, and takes its food as usual, until a short time before death. *The state of illness may last only a very few hours, and in some cases it is not observed at all.*

In a very large number of the experiments included in Table III. (B), the results are negative, and the animals suffer no inconvenience whatever from the inoculation. *These experiments are therefore entirely painless.*

*In the event of pain ensuing as the result of an inoculation, a condition attached to the license requires that the animal shall be killed under anæsthetics as soon as the main result of the experiment has been attained.*

*With respect to the experiments included in Table III. (B), attention may especially be directed to the large numbers performed for the preparation of remedies, and on behalf of various public authorities.* Five licensees performed 3,857 inoculation experiments for the testing of anti-toxins. Seventeen licensees report together 3,997 inoculation experiments, nearly the whole of which were performed for official bodies, including the Local Government Board, the House of Commons Ventilation Committee, the Royal Commission on Tuberculosis, the Board of Agriculture, several county councils, and many municipal corporations. The experiments comprised a very large number for the purpose of testing milk for tuberculosis, others for ascertaining whether hair is infected with anthrax, for the examination of sewage and atmospheric air, and the like. Numerous inoculations are required also for the diagnosis of disease in man and animals; and many are performed in the course of the cancer investigations, now in progress in several places.

The number of injections made during the year 1902 for the diagnosis of rabies in dogs is 153.

During the year the usual inspections of registered places have been made by Sir James Russell and myself, and have been severally reported. We have found the animals everywhere suitably lodged and well cared for, and the licensees desirous of acting in every way in accordance with the letter and the spirit of the Act. The following are the only irregularities that have occurred:—

In three instances licensees holding Certificate A for the performance of inoculation experiments without anæsthetics, thinking that the certificate ran for the same period as the license, performed such experiments after the date on which the certificate expired. In each case the licensee was cautioned.

A licensee, who held Certificates A and E for the performance of inoculation experiments on cats and dogs without anæsthetics, made certain inoculations into guinea-pigs and rabbits which were anæsthetised. It must be again pointed out that when an anæsthetic is used for the performance of any operation, and the animal allowed to recover therefrom, the certificate required by the Act to cover the proceeding is that known as B, and not A.

I have the honour to be, Sir,

Your obedient servant,

G. D. Thane, Inspector.

The Right Hon. Aretas Akers Douglas,
His Majesty's Principal Secretary of State.

---

Let us look at the plain facts of this Report. Six out of seven experiments are of the nature of inoculations. It is these, *and these alone*, that are made without anæsthetics. In a very large number of them, there is no result, and no possibility of pain. In some, there is that pain which would come of the same disease in the course of Nature. The majority of these inoculations are made on behalf of Government, or of Councils and Corporations, in the direct service of the public health: others are made for cancer research, others for the diagnosis of disease in man and animals. The animals under Certificate B (or B + EE) may be compared with a like number of like animals after operation, under anæsthesia, by a veterinary surgeon, with this difference, that many of them lose health, or suffer some disablement, and so die, or are killed. But whatever is *done*, must be done under anæsthesia; nothing must be done, either at the time or afterward, that could cause pain without anæsthesia. Some of them might be compared to pet animals kept alive throughout the distress and infirmities of old age. Anyhow, they cannot be compared with a like number of sheep and cattle mutilated, without anæsthesia, by farmers and breeders: nor with a like number of pheasants and rabbits wounded, without anæsthesia, in sport. "The sportsman can plead, in excuse, only his right to please himself and his friends in his own way, and his intention to inflict, for his own pleasure, not 'torture,' but only death. Experiments on animals have this excuse, that they are necessary not only for science but also for practice. In physiology, and in pathology, and in the prevention and the cure of disease, and in the operations of surgery, they have helped to save human lives literally in thousands and tens of thousands. Admit, that some of them involve pain, some have no direct bearing on practice, some fail, or are misinterpreted; there remains a whole legion of ourselves, rescued from disease and death, a multitude past all reckoning and ever increasing."

